# Current News

**Published:** 1905-07

**Authors:** 


### Current News.

### LEWIS AND CLARK DENTAL CONGRESS.

Following is the programme of the Lewis and Clark Dental Congress, to be held at Portland, Ore., July 17, 18, 19, and 20, 1905:

MORNING.

9 to 12. Exhibits.

10.00. Meeting of General Committee.

10.30. Salutation, Norris R. Cox, Chairman General Committee.

Address of welcome, Harry Lane, M.D., Mayor of Portland.

Response.

Organization and election of officers.

AFTERNOON.

Papers, clinics, exhibits. To follow on July 17, 18, 19, 20, as arranged later. Programme to be completed at 5 P.M., July 20.

Evening of July 20, Congress adjourns to "The Trail," Lewis and Clark Fair.

ESSAYS.

(Not complete.)

I. "Pathology." John Endleman, Philadelphia Pa. Discussion opened by B. S. Scott, Tacoma, Wash.

II. Symposium on Empyema of the Maxillary Sinus (ten-minute papers):

1. "The Anatomy of the Maxillary Sinus and the Relation of the Superior Teeth to the Sinus." Dr. James G. Sharp, San Francisco, Cal.

2. "The Etiology and Diagnosis of Empyema of the Maxillary Sinus." Dr. Frank L. Platt, San Francisco, Cal.

3. "The Surgical Treatment of the Maxillary Sinus." Dr. John S. Marshall, U.S.A.

Symposium on Fractures of the Mandible (ten-minute papers):

1. "The Anatomy of the Mandible, with Special Reference to the Muscles which control its Movements and the Localities at which Fractures are most liable to occur." Dr. James G. Sharp, San Francisco, Cal.

2. "The Classification of Fractures of the Mandible, and their Diagnosis." Dr. William A. Bryant, San Francisco, Cal.

3. "The Surgical Treatment of Fractures of the Mandible, Suturing, etc." Dr. John S. Marshall, U.S.A.

4. "The Mechanical Treatment of Fractures of the Mandible. Interdental Splints, External Splints, Fracture Bands, etc." Dr. Charles H. Bowman, San Francisco, Cal.

III. "Why are not All Successful?" Dr. J. C. Hennessy, Reno, Nev. Discussion opened by D. J. Wait, Helena, Mont.

IV. "Mouth Breathing: Its Relation to Orthodontia." (Eighteen lantern slides.) Ray D. Robinson, Los Angeles, Cal. Discussion opened by E. G. Howard, Los Angeles, Cal.

V. "Dental Lesions of Animals—Domestic, Captive, and Wild." (Lantern slides.) William Bebb, Los Angeles, Cal. Discussion opened by Arthur D. Black, Chicago, Ill.

VI. "History of Dentistry." (Lantern slides.) Burton Lee Thorpe, St. Louis, Mo. Discussion opened by Arthur W. Chance, Portland, Ore.

VII. "Accidental Cleft Palates." H. J. Janlusz, New York, N. Y. Discussion opened by J. A. Meyer, Tacoma, Wash.

VIII. "The Status of the Inlay: Gold and Porcelain." C. N. Thompson, Chicago, Ill. Discussion opened by B. F. Eshleman, Tacoma, Wash.

IX. "Interstitial Gingivitis." Eugene S. Talbot, Chicago, Ill. Discussion opened by G. V. I. Brown, Milwaukee, Wis.

X. Ether Spray as an Obtundent." Crittenden Van Wyck, Oakland, Cal. Discussion opened by William A. Bryant, San Francisco, Cal.

XI. "Dental Standards." Clyde Payne, San Francisco, Cal. Discussion opened by William A. Cumming, Portland, Ore.

XII. "Discrimination in the Selection of Filling-Material." C. N. Johnson, Chicago, Ill. Discussion opened by John S. Marshall, San Francisco, Cal.

XIII. Subject not announced. G. V. I. Brown, Milwaukee, Wis. Discussion opened by Eugene S. Talbot, Chicago, Ill.

XIV. "Pulp Technique." M. L. Rhein, New York, N. Y. Discussion opened by C. N. Johnson, Chicago, Ill.

XV. "A Plea for the Left Hand." F. H. Metcalf, Sacramento, Cal. Discussion opened by S. J. Barber, Portland, Ore.

XVI. "The Needs of Reform in Dental Education." D. D. Smith, M.D., D.D.S., Philadelphia, Pa. Discussion opened by ——.

XVII. Not completed.

XVIII. "Local Anæsthesia," or "Dental Education." W. G. Wyckoff, Philadelphia, Pa. Discussion opened by ——.

XIX. "Diagnostic and Therapeutic Uses of X-Rays in Dentistry and Oral Surgery." (With lantern slides.) M. I. Schamberg, Philadelphia, Pa. Discussion opened by

CLINICS AND CLINICIANS.

1. M. L. Rhein, New York, N. Y. "Technique of Pulp Removal and Root-Canal Cleansing, and packing Gold by Means of Electro-Magnetic Mallet."

2. Morris T. Schamberg, Philadelphia, Pa. "Root Amputation *versus* Ablation for the Cure of Chronic Apical Abscesses."

3. C. N. Thompson, Chicago, Ill. "Gold Inlay." Table Clinic: "Porcelain Inlays."

4. G. V. I. Brown, Milwaukee, Wis. Surgical clinic.

5. R. B. Gentle, New York, N. Y. "Cavity Preparation for Porcelain Inlays."

6. Alice M. Steeves, Boston, Mass. "Useful Root-Canal Filling."

7. P. M. Wuillemin, San Francisco, Cal. "Extraction under Nitrous Oxide Anæsthesia."

8. F. R. Sandusky, Nashville, Tenn. "Saddle Bridge-Work, Molars supplied, using all Porcelain Dummies, with Porcelain Crowns for Anchorage."

9. R. C. Brophy, Chicago, Ill. (*a*) "Porcelain Work without Use of Electricity." (*b*) "Metalo-Plastic Plate-Work."

10. Ray D. Robinson, Los Angeles, Cal. "Appliances for retaining Teeth after shifting Occlusion."

11. J. M. Yates, Portland, Ore. "Administration of Somnoform."

12. J. L. Pease, Oakland, Cal. Subject to be announced.

13. F. W. Hergert, Seattle, Wash. "Swaging Metal Plates by the Holmes-Palmer Method."

14. O. J. Fruth, St. Louis, Mo. "Raising the Bite by Means of Gold Inlays and Method of making the Same."

15. F. L. Platt, San Francisco, Cal. "Porcelain Inlays in Crown- and Bridge-Work."

16. B. F. Eshelman, Tacoma, Wash. "Something Interesting to Porcelain Workers."

17. C. B. Reynolds, Seattle, Wash. "Use of Cohesive and Non-Cohesive Gold in Molar."

18. J. S. Engs, Oakland, Cal. "Photo-Micrographs."

19. J. S. Baldridge, Wooley, Wash. "The Restoration of an Incisal Angle with High-Fusing Porcelain."

20. H. J. Smith, Genesee, Idaho. "Removal of Deposits and Stains from Teeth of Adults and Children, with Medical Applications Before and After."

21. H. W. Bates, Denver, Col. "Simple Manner of making Dies and Swaging Plates from Plaster Impressions."

22. A. M. Baker, San Jose, Cal. Subject to be announced.

23. G. W. Schwartz, Chicago, Ill. "Removable Bridges."

24. F. K. Ledyard, San Jose, Cal. Subject to be announced.

25. C. L. Rose, Fargo, N. D. "Gold Inlay."

26. J. H. Merritt, Oakland, Cal. "Restoration of Incisal Angle, using Jenkins Porcelain."

27. A. M. Magee, Louisiana, Mo. "Some Difficult Things presenting, and what We will do with Them."

28. W. V. B. Ames, Chicago, Ill. "Adaptation of Entire Artificial Dentures."

29. C. A. Southwell, Boise, Idaho. "Filling Molar-Appro-Mesial Cavity with Gold, using Electric or Automatic Mallet."

30. F. W. Lawrence, St. Louis, Mo. "Something in Porcelain."

31. C. N. Johnson, Chicago, Ill. "Demonstrations of Cavity Preparation on Models." (Table clinic.)

32. R. A. Rawlings, Bismarck, N. D. "Filling Disto-Occlusal Cavity in Upper Bicuspid with Gold."

33. William Bebb, Los Angeles, Cal. "Anatomical Specimens." (Table clinic.)

34. William H. DeFord, Des Moines, Iowa. "Demonstrations on Somnoform."

35. Jessie R. DeFord, Des Moines, Iowa. "Demonstrations of Somnoform."

36. C. S. Irwin, Vancouver, Wash. Table and Chair Clinic, using Tin.

37. E. R. Tait, Oakland, Cal. "Filling Teeth with Plastic Mat Gold."

38. J. W. Nebett, Chicago, Ill. "Showing Method of swaging with Water; also, Filling Matrix with Porcelain in a Manner that will overcome Warping."

39. V. H. Frederick, St. Louis, Mo. "Continuous Gum Work."

40. E. B. Edgers, Seattle, Wash. "Treating Putrescent Pulp-Canals by the Use of Sodium and Potassium Compound and filling immediately."

41. L. P. Haskell, Chicago, Ill. "Continuous Gum Work."

42. A. J. Holmes, New Westminster, B. C. "Simple Method of constructing Richmond Crown."

43. Crittenden Van Wyck, San Francisco, Cal. "Showing Van Wyck Obtunder and Practical Demonstrations with Same."

44. B. F. Eshelman, Tacoma, Wash. "Something Interesting to Porcelain Workers."

45. George N. Wasser, Cleveland, Ohio. "High-Fusing Porcelain Inlay."

46. W. J. Hacking, New Westminster, B. C. "Technique of an Accurate Fitting and Articulated Seamless Crown."

47. H. Russell Hill, Hamilton, Mo. "Open Face Contour Crown-Work."

48. Los Angeles Porcelain Club (seven in number). Seven men will work porcelain—a continuous clinic.

49. W. N. Murray, Minneapolis, Minn. "Gold Inlay."

50. Valentine K. Irion, New Orleans, La. "A Full Porcelain Bridge without Use of Furnace or Baking" (the Lowque system).

51. A. P. Johnstone, Anderson, S. C. "A New and Original Way of fitting Porcelain Crowns, Incisors, Cuspids, Bicuspids, and Molars, Upper or Lower, to their Respective Roots." (Table clinic.)

52. Fred H. Metcalf, Sacramento, Cal. "Positions at the Chair for Ambidextrous Operator."

53. G. M. Osterberg, Seattle, Wash. "Adaptation of the Davis Crown by baking."

54. G. Maurice Crow, Los Angeles, Cal. "The Use of Porcelain Stains."

55. C. F. Sweet, Minot, N. D. "Gold Fillings in Porcelain Teeth."

56. W. A. Cummings, Portland, Ore. Subject to be announced.

57. J. J. F. McLaughlin, North Adams, Mass. "Rowan's Extra Pliable Gold Rolls and Rolled Gold for Contour Fillings."

58. E. DeWitt R. Garden, Tarrytown, N. Y. "Mineral Plate Work."

59. J. G. Hollingsworth, Kansas City, Mo. "Demonstrating New Reinforcement for Porcelain Crowns." (Table clinic.)

60. Portland Porcelain Club (six in number).

61. D. D. Smith, Philadelphia, Pa. "Exhibition of the Correct Method of filling Front Teeth."

62. N. R. Cox, Portland, Ore. "Somnoform Demonstrations."

63. D. I. Wadsworth, Portland, Ore. "Gold Inlay showing Swaging Device." (Table clinic.)

64. George D. Peters, Portland, Ore. "Removable Crown- and Bridge-Work." (Peeso's method.)

65. John S. Marshall, U.S.A., Presidio, Cal. Surgical Clinic.

66. E. A. Tripp, Salt Lake City, Utah. "Impressions of Cleft Plate and Obturator."

67. Mark Hayter, Dallas, Ore. "Backing Porcelain Facings."

68. J. B. Burns, Payette, Idaho. "Practical Cases of Removable Upper Partial Plate, with Attachments." "Deposits on Teeth, and Effect." (Table clinic.)

69. L. A. Stemmly, Myrtle Point, Ore. (*a*) "Method of backing Facings." (Table demonstration.) (*b*) "Method of placing Fillings in Porcelain Crowns."

70. J. R. Cardwell, Portland, Ore. "Trimming Model for Vacuum Plates."

71. W. N. Murray, Minneapolis, Minn. "Gold Inlay."

72. W. G. Wyckoff, Philadelphia, Pa. "Painless Extraction with Local Antiseptic."

73. E. L. Hutchinson, Honolulu, S. I. "Gold Inlay."

74. F. E. Roach, Chicago, Ill. "A New Fusible Cement."

75. J. S. Kirkwood, Salt Lake City, Utah. "Extraction of Teeth and Live Pulps by Use of Somnoform."

76. Charles W. Day, Vinita, Ind. Ter. "Invisible Attachments for Fixed or Removable Bridges."

77. Frank N. Walgamott, Portland, Ore. "Hypnotism and its Use in Dentistry." (Demonstration.)

78. J. R. Entrikin, Des Moines, Iowa. "The Filling of Root-Canals with other than the Ordinary Chloropercha."

79. R. Siddall, The Dalles, Ore. "Extracting Teeth, showing Use of Elevator."

80. W. F. Mack, Salt Lake City, Utah. "A New Idea in a Porcelain Crown."

81. D. T. Hill, Syracuse, Neb. Dental Crown and Plate Anchor."

82. D. O. M. LeCron, St. Louis, Mo. "Porcelain and its Correct Fusing 'Table.'"

83. H. N. Smith, Seattle, Wash. "Method of making Seam Crowns with Smith's Contour Pliers."

84. S. M. Hampton, Helena, Mont. Gold Filling."

85. D. J. Wait, Helena, Mont. "Use of Vernon's Gold for filling."

86. A. F. Merriman, Oakland, Cal. "Gold Filling."

87. O. D. Ireland, Portland, Ore. "Articulation of Teeth after Bonwill."

88. Dr. Stratford, New York, N. Y. "Somnoform Demonstration."

89. Lelan Otis Green, Chicago, Ill. "Painless Extraction under Influence of the Local Anæsthetic Acestoria."

90. E. W. Dodez, Ft. Wayne, Ind. "The Filling of Root-Canals with Oxpara."

LIST OF EXHIBITORS.

S. S. White Dental Manufacturing Company, Philadelphia, Pa. Ritter Dental Manufacturing Company, Rochester, N. Y. L. D. Caulk Company, Philadelphia, Pa. Gideon Sibley, 1314 Filbert Street, Philadelphia, Pa. Klewe & Co., Portland, Me. Boston Pharmacal Company, San Francisco, Cal. The J. W. Edwards Company, San Francisco, Cal. W. V. B. Ames, 36 Washington Street, Chicago, Ill. Buffalo Dental Manufacturing Company, Buffalo, N. Y. J. W. Ivory, 51 N. Tenth Street, Philadelphia, Pa. Detroit Dental Manufacturing Company, Detroit, Mich. Dr. X. Dodal, 1307 Howard, San Francisco, Cal. Edward Rowan, 831–7 East 163d Street, New York, N. Y. Dr. L. O. Green, 100 State Street, Chicago, Ill. C. H. Pinches, 1181 Broadway, New York, N. Y. Keasby Mattison Company, Ambler, Pa. Hisey Dental Manufacturing Company, St. Louis, Mo. Chicago Dental Specialty Company, Chicago, Ill. The American Cabinet Company, Two Rivers, Wis. The Rasom & Randolph Company, Toledo, Ohio. Lambert Pharmacal Company, St. Louis, Mo. Armour & Co., Chicago, Ill. Kress & Owen Company, Chicago, Ill. A. P. Gould & Co., Canton, Ohio. Dr. J. C. Graft, Newark, N. J. Harvard Dental Manufacturing Company, Canton, Ohio. A. C. Clark & Co., 21 East Randolph Street, Chicago, Ill. Blair Manufacturing Company, Louisville, Ky. Lee S. Smith & Son, 1301–3 Arch Street, Philadelphia, Pa. J. M. Ney & Co., 265 Asylum Street, Hartford, Conn. Dentists' Supply Company, 109 West 42d Street, New York, N. Y. E. DeTrey & Son, Philadelphia, Pa. Electro-Dental Manufacturing Company, 1228 Cherry Street, Philadelphia, Pa. Cleveland Dental Manufacturing Company, Cleveland, Ohio. Horlick Food Company, Racine, Wis. Oakland Chemical Company, New York, N. Y. Hall & Ruckel, New York, N. Y. Victor Electric Company, 56–61 Market Street, Chicago, Ill. Florence Manufacturing Company, Florence, Mass. E. C. Moore & Co., 112 Miami Avenue, Detroit, Mich. B. C. Smith & Son, Pittsburg, Pa. A. J. Watts & Co., 127 East 23d Street, New York, N. Y. Sanitol Chemical Company, St. Louis, Mo. Randall-Faichney & Co., Boston, Mass. Tenax Dental Compound Company, Terra Haute, Ind.

This programme is not complete up to date. The entire programme will be completed by July 1.

Kindly call attention to the location of a sub-station of the Portland Post-Office in the meeting of the Congress. Dentists should have their mail addressed to Lewis & Clark Dental Congress, Armory Oregon National Guard, Portland, Ore.

The social programme of the Congress will be largely of an informal character. The Stomatological Club of Portland has arranged for club-rooms in the Armory Building, the meeting-place of the Congress, where informal social gatherings will take place each day.

The Congress invites its members to participate in all the exhibitions and shows on the Trail of the Lewis & Clark Fair for the final entertainment following the Congress, on the night of July 20.

---

### NATIONAL DENTAL ASSOCIATION, SECTION II.

Following is the corrected programme for Section II. of the National Dental Association:

Dr. J. V. Conzett, Dubuque, Iowa, "Gold as a Filling-Material."

Dr. B. L. Thorp, 3666 Olive Street, St. Louis, Lantern Lecture: "Pioneer Manipulators of Gold-Foil."

Dr. Chas. Milton Ford, 523 West One Hundred and Forty-first Street, New York, "Dental Education."

Dr. W. R. Clack, Clear Lake, Iowa, "The Necessity for a Method of preserving the Integrity of the Interproximal Space."

Dr. D. O. M. LeCron, 501 Missouri Trust Building, St. Louis, "A Few Experiments in Porcelain."

Dr. D. W. Fellows, Portland, Me., "A Century of Standard Dental Writings."

Dr. B. Holly Smith, 1007 Madison Avenue, Baltimore, "Operative Dentistry."

Dr. S. H. Guilford, 1631 Walnut Street, Philadelphia, "The Nomenclature of Orthodontia."

Dr. Wm. H. Potter, 16 Arlington Street, Boston, Mass., "The Use of the Summer Vacation in the Education of the Dental Student."

Dr. I. J. Wetherbee, 120 Boylston Street, Boston, Mass., "Tin-and-Gold: Its Possibilities and Uses as a Filling-Material."

Dr. H. E. Roberts, *Chairman*,
Philadelphia, Pa.
Dr. C. S. Butler, *Secretary*,
Buffalo, N. Y.

---

### CLINIC SECTION OF THE NATIONAL DENTAL ASSOCIATION.

The work of the Clinic Section is progressing most favorably. Everything at present indicates that the operative clinic will be the largest that the National Dental Association has ever held. There will be forty operators for both mornings upon which the clinics will be held. The territory from Maine to Utah and from Minnesota to Texas has been very fully covered, and men from almost all of the States in the section named have signified their willingness to be present and operate. The majority of the men of the G. V. Black Dental Club will be present and will operate upon both days. Such well-known Northwestern men as Drs. Searl, Lewis, Clack, Conzett, Beemer, G. D. Moyer, W. H. K.

Moyer, W. D. James, F. S. James, Gallagher, Carlson, Fawcett, etc., will once more operate in a body, as was done at the International Dental Congress.

Dr. T. W. Brophy, of Chicago, has kindly consented to assist and will make a surgical operation. Dr. Brophy's clinics are of such a high order that those interested are certain of seeing something which they will not soon forget.

The well-known Dr. M. E. Smith, of Lynn, Mass., will also make a surgical operation.

Somnoform and narcotile will be very fully demonstrated.

A large number of gentlemen will give table clinics.

At the present writing I feel confident in saying that the best men in the profession will operate at Buffalo on July 26 and 27. A full report has not been received from all the men on the committee, but sufficient data are before me upon which to base the opinion above expressed.

E. K. Wedelstaedt,
*Secretary Clinic Section.*

New York Life Building, St. Paul, Minn.

---

### MAINE DENTAL SOCIETY.

The Fortieth annual meeting of the Maine Dental Society will be held at Portland, Me., July 18, 19, and 20, 1905.

George E. Dow,
*Chairman Executive Committee.*

---

### GRADUATES OF THE KANSAS CITY DENTAL COLLEGE.

Will you kindly forward your address to me, so as to complete a roster of the graduates? Don't take it for granted your address is known.

J. P. Root.

Deardorff Building, Kansas City, Mo.

### FARGO DISTRICT DENTAL SOCIETY.

There has been organized the Fargo District Dental Society, at Fargo, North Dakota, which meets the third Monday of every month. The following officers were elected:

President, S. J. Hill; Vice-President, H. L. Starling; Secretary, Albert Hallenberg; Treasurer, C. L. Rose.

*Executive Committee.*—L. C. Davenport, J. L. Graves, F. A. Bricker.

Albert Hallenberg,
*Secretary.*

### SOUTH DAKOTA STATE BOARD OF DENTAL EXAMINERS.

The next meeting of the South Dakota State Board of Dental Examiners will be held at Mitchell, S. D., July 11, beginning at 1.30 p.m. All candidates will be required to do practical work in both operative and prosthetic dentistry, and should bring all instruments and materials necessary to do the same. Vulcanizer, lathe, and swaging appliances will be furnished by the board. Application, together with fee of ten dollars, must positively be in the hands of the Secretary before July 7.

G. W. Collins.

### "F. D. I." INTERNATIONAL DENTAL FEDERATION.

The next annual meeting of the Executive Council of the Fédèration Dentaire Internationale will convene in Hanover, Germany, August 7, 1905, immediately following the annual meeting of the Central-Verein Deutscher Zahnarzte. Announcement of the programme for the meeting and the projected work for the Federation during the present period will shortly be made through the dental journals and through the official bulletin of the Federation.

Edward C. Kirk,
*Secretary-General.*

